# Using artificial intelligence and predictive modelling to enable learning healthcare systems (LHS) for pandemic preparedness

**DOI:** 10.1016/j.csbj.2024.05.014

**Published:** 2024-05-17

**Authors:** Anshu Ankolekar, Lisanne Eppings, Fabio Bottari, Inês Freitas Pinho, Kit Howard, Rebecca Baker, Yang Nan, Xiaodan Xing, Simon LF Walsh, Wim Vos, Guang Yang, Philippe Lambin

**Affiliations:** a Department of Precision Medicine, GROW School for Oncology, Maastricht University Medical Centre+, Maastricht, the Netherlands; b Radiomics (Oncoradiomics SA), Liege, Belgium; c CDISC, Austin, TX, United States; d National Heart and Lung Institute, Imperial College London, London, United Kingdom; e Bioengineering Department and I-X, Imperial College London, London, United Kingdom

**Keywords:** Learning Healthcare Systems, Artificial Intelligence, Predictive Modeling, Data Harmonization, Explainable AI

## Abstract

In anticipation of potential future pandemics, we examined the challenges and opportunities presented by the COVID-19 outbreak. This analysis highlights how artificial intelligence (AI) and predictive models can support both patients and clinicians in managing subsequent infectious diseases, and how legislators and policymakers could support these efforts, to bring learning healthcare system (LHS) from guidelines to real-world implementation. This report chronicles the trajectory of the COVID-19 pandemic, emphasizing the diverse data sets generated throughout its course. We propose strategies for harnessing this data via AI and predictive modelling to enhance the functioning of LHS. The challenges faced by patients and healthcare systems around the world during this unprecedented crisis could have been mitigated with an informed and timely adoption of the three pillars of the LHS: Knowledge, Data and Practice. By harnessing AI and predictive analytics, we can develop tools that not only detect potential pandemic-prone diseases early on but also assist in patient management, provide decision support, offer treatment recommendations, deliver patient outcome triage, predict post-recovery long-term disease impacts, monitor viral mutations and variant emergence, and assess vaccine and treatment efficacy in real-time. A patient-centric approach remains paramount, ensuring patients are both informed and actively involved in disease mitigation strategies.

## Graphical Abstract

**Figure:**
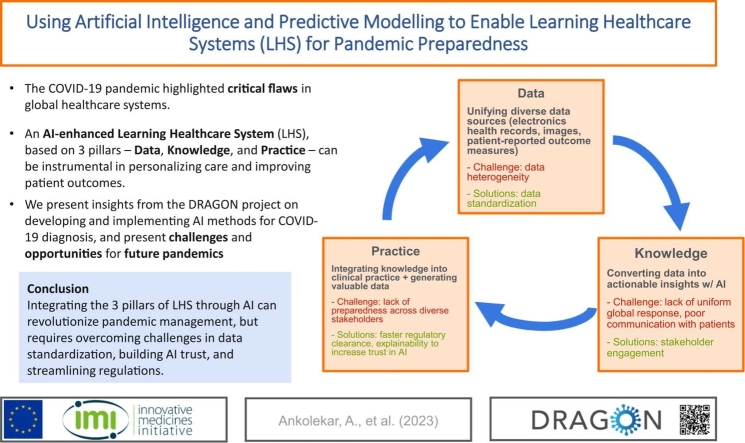

**Summary Box**

*The COVID-19 pandemic exposed weaknesses in health care systems around the world in the prevention and response to large-scale epidemics and pandemics. Challenges included shortages of medical supplies, overwhelmed healthcare systems, along with lack of evidence-based decision making and misinformation.*

*A learning healthcare system (LHS) that leverages big data and artificial intelligence (AI) could be crucial for effective future pandemic management. To achieve a fully integrated LHS, data standardization, harmonization and sharing is fundamental. Distributed learning along with data harmonization represent a viable solution to these challenges.*

*AI techniques like machine learning (ML) and natural language processing could be instrumental in unlocking knowledge from data. AI can aid in diagnosis, risk stratification, and prediction of outcomes. Effective communication with stakeholders is essential for translating knowledge into action.*

*Healthcare systems must be ready to implement AI-driven insights, and also be able to access fast-track regulatory approvals for medical devices and guidelines. Building trust in AI through explainable AI (XAI) is crucial for widespread adoption.*

*Overall, integrating data, knowledge, and practice through AI and an LHS can greatly improve pandemic management, but challenges like data standardization, trust in AI, and regulatory processes need to be addressed.*

## 1 Introduction

Since the first report of the then-unknown illness in December 2019, the coronavirus disease (COVID-19) pandemic has presented the world with unprecedented challenges and exposed many weaknesses in the way we are able to detect potential pandemics early on, identify new pathogens and learn from the events as they transpire [1], [2]. Some of the biggest challenges were shortages of medical supplies and healthcare systems that were quickly overwhelmed, a lack of data on the spread of COVID-19 and its impact on healthcare systems which made it difficult for healthcare providers and policymakers to respond effectively, along with a lack of accurate information for the general public [3], [4].

This paper emerges from the collective efforts of DRAGON (RapiD and SecuRe AI enhAnced DiaGnosis, Precision Medicine and Patient EmpOwerment Centered Decision Support System for Coronavirus PaNdemics), a collaborative project bringing together researchers, clinicians, technology developers, patients, and patient representatives to harness artificial intelligence (AI) in creating a more responsive and adaptive healthcare system. The DRAGON consortium has focused on conceptualizing and implementing AI-driven strategies to navigate the complex landscape of pandemic data, extract meaningful insights, and translate these into actionable policies and practices. Our findings during the COVID-19 pandemic suggest that the future of healthcare lies in the ability to learn and rapidly adapt using AI and predictive modeling.

In this review, we aim to present a consolidated view of the lessons learned from the COVID-19 pandemic, underscored by the DRAGON consortium's findings and other pivotal research, culminating in a set of forward-looking recommendations. We advocate for a Learning Healthcare System (LHS) that not only capitalizes on the vast quantities of clinical and surveillance data generated during such crises but also prioritizes patient-centric approaches in preparing for and managing future pandemics.

While the COVID-19 pandemic was not the first to affect the world in such magnitude, it was the first to occur in the modern world, where long and short-distance travel is more accessible than ever, making it extremely difficult to contain the virus. Moreover, during this pandemic, an amount of data never seen before was generated, including clinical data (patient demographics, medical history, lab results, symptoms, imaging) and surveillance data (confirmed cases, hospitalizations, deaths) [5]. The cornerstone of a new approach to healthcare management and patient-centric medicine should leverage this vast amount of information and data, which should be gathered, harmonized, shared and holistically combined in a learning healthcare system [6].

Through the lens of the DRAGON consortium's endeavors, this paper reviews the complexities of integrating AI into healthcare frameworks. To be able to do this, we look in retrospect at the acute phase of the COVID-19 pandemic and analyse both negative and positive aspects and outcomes of its management and control. We chart a path forward, outlining how these advanced technologies can be harnessed not just to manage the current pandemic's aftermath but to proactively shape a robust response to future health crises.

## 2 Methodology

This narrative review synthesizes diverse insights to assess the role of AI and predictive modeling in pandemic preparedness. Insights were derived from a comprehensive review of literature and expert discussions, although not through a systematic review process. Key sources included academic databases, reports from health organizations, and discussions from the DRAGON consortium's workshops which included researchers, patients, and professionals across the healthcare and technological spectrum.

### 2.1 Data sources and selection

Peer-reviewed literature was sourced from academic databases including PubMed, Scopus, and Google Scholar. The selection prioritized articles detailing AI applications in healthcare, particularly those related to pandemic contexts. Additional sources included authoritative reports from health organizations such as the World Health Organization (WHO), which provided contextual understanding of the pandemic's scope and the healthcare system's response. The literature was chosen based on relevance to AI's role in healthcare during the COVID-19 pandemic, with an emphasis on contributions that could inform strategies for future health crises.

### 2.2 Contribution of the DRAGON consortium

Discussions and findings from the DRAGON consortium's workshops significantly contributed to the thematic development of this review. The consortium's focus on integrating AI into practice for improved pandemic response, as well as the experiences of various partners, provided critical insights and a practical framework for analyzing the literature. These discussions aided in identifying both the potential and the challenges of employing AI and predictive modeling in real-time health crisis management and in retrospective analyses.

### 2.3 Choice of framework

We chose the Learning Healthcare System (LHS) as a guiding model to structure our narrative review. The LHS originated from the Institute of Medicine's seminal report and describes a healthcare system that continuously learns from patient data and experiences to improve patient outcomes, increase efficiency, and reduce costs [7], [8], [9]. It integrates data from multiple sources, including electronic health records (EHR), clinical trials, and patient-generated data, to inform clinical decision-making and guide continuous improvement.

Our choice of the LHS over other potential frameworks such as the eHealth framework and the WHO Health Systems Resilience framework is because the LHS is predicated on the notion that data from patient care experiences and research should be systematically integrated to inform and improve future healthcare decisions and outcomes. It emphasizes a cycle of continuous learning, facilitated by advancements in AI and predictive analytics, both of which are particularly relevant in the rapidly evolving context of a pandemic. In contrast, while the eHealth framework underscores the role of information and communication technologies in health, and the WHO framework addresses systemic resilience, the LHS framework uniquely aligns with the aim of this review—to critically evaluate and recommend strategies for real-time, data-driven pandemic preparedness and response. This focus on dynamic learning and adaptability makes it most suitable for exploring how AI can be harnessed to create a proactive and learning-oriented healthcare ecosystem.

### 2.4 Data analysis

**Fig. 1:**
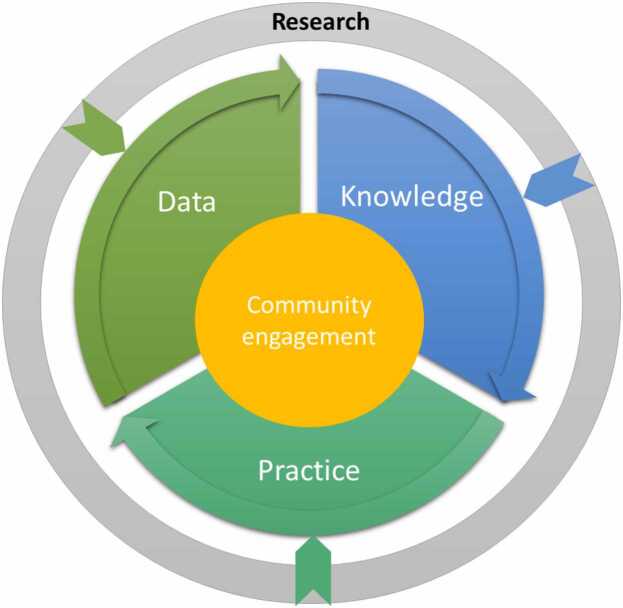
**:** The Learning Healthcare system cycle.

The literature was synthesized to highlight the three key pillars of LHS—Data, Knowledge, and Practice. Fig. 1, which will be referenced and described in detail in the subsequent section, illustrates the LHS framework operationalized within the context of our review. This framework visualizes the cyclical and dynamic nature of the LHS, where the key pillars are the drivers of a continuous learning process, centered around community engagement. In the figure, 'Data' represents the empirical foundation, consisting of raw and processed patient-related information. In the medical domain, such data are characterized by their volume, velocity, variety, and the need for veracity—collectively known as the 'four Vs' of big data [10]. This dimension emphasizes the importance of data standardization and interoperability necessary for integrating diverse data sets. 'Knowledge' in the diagram is depicted as the insights derived from data through the application of AI and analytics and represents the transformation of data into a format that healthcare professionals and policymakers can use for evidence-based decisions. Finally, 'Practice' represents the implementation of this knowledge into the healthcare delivery system, encompassing the development and application of clinical guidelines, patient care protocols, and health policies. At the core of the model, community engagement signifies the partnership between patients, healthcare providers, and the public, which is vital for the success of an LHS.

Data gathering, literature analysis and synthesis were collaboratively executed by AA, LE, and FB. To minimize the inherent biases of narrative reviews, each researcher conducted independent evaluations of sources to ensure a thorough cross-validation of the findings. Regular critical discussions were held to reconcile findings and consolidate consensus viewpoints, in order to mitigate selection and confirmation biases. IP oversaw the structured approach and helped maintain methodological integrity throughout the review process. Specialized insights were integrated from KH and RB on data standardization, as well as from YN, XX, GY, and SLFW. on federated learning and predictive analytics to ensure a multidisciplinary approach and reducing the risk of individual biases skewing the review. This collective effort, incorporating feedback from multiple disciplines, served to mitigate individual bias and reflect a consensus view within the consortium.

## 3 COVID-19 pandemic overview – challenges and opportunities in the management of COVID-19 pandemic

**Fig. 2:**
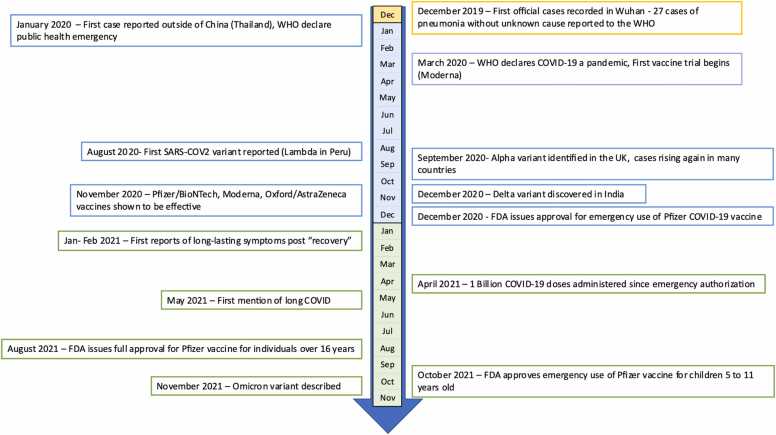
**:** COVID-19 pandemic timeline.

The COVID-19 pandemic started in late 2019 in Wuhan, China, with the first cases of pneumonia of unknown cause reported to the World Health Organization. The novel coronavirus, later named SARS-CoV-2, was identified as the source of these infections. The virus continued to spread, and by March 2020, the WHO declared COVID-19 a pandemic [11]. Healthcare systems globally were operating at maximum capacity with resources being redirected away from the standard of care procedures. During the initial stages of the outbreak, two primary variants of SARS-CoV-2, lineage A and lineage B, were identified. As the virus evolved, it accumulated key mutations, notably D614G in the spike protein and P323L in NSP12 by March 2020. These mutations led to the emergence of the B1 lineage, which saw widespread global dominance. While there was a drop in infection rates during the summer of 2020, a resurgence, often referred to as the "second wave", was observed in late summer/early fall, during which other notable variants started to be reported [12]. In November 2020, data from vaccine clinical trials were released, showcasing the safety and efficacy of several vaccine candidates. Among these were two mRNA vaccines, COMIRNATY and Spikevax, as well as the AstraZeneca's viral vector vaccine, Vaxzervia. The latter, in particular, played a pivotal role in the UK's vaccination campaign, substantially aiding in mitigating the pandemic's impact in the region [13]. Emergency use authorization by the U.S. Food and Drug Administration (FDA), European Medicines Agency (EMA), Medicines and Healthcare products Regulatory Agency (MHRA), WHO and other regulatory agencies followed shortly after vaccine campaigns globally progressed, and by April 2021, 1 billion doses were administered. Concurrently, long-lasting symptoms, the so-called “long-COVID” emerged as a challenge to patients and doctors, for which there is no known cure [14]. Many different therapeutics were evaluated for the treatment of acute COVID-19, including repurposing of existing drugs, and some have shown promise in early studies, while others showed no clinical benefit. The major landmarks of the pandemic up to the end of 2021 are depicted in Fig. 2.

As outlined above there were many challenges in the management of the global COVID pandemic. The main issues faced by patients and healthcare providers around the world can be summed up following the three main pillars of the LHS: Knowledge, Data and Practice (see Table 1.).

**Table 1:** Challenges and opportunities in the management of the COVID-19 pandemic.

LHS pillars	Challenge	Source of challenge	Opportunities
Data	No data standardization	Lack of awareness of available standards. Difficulties (real and perceived) in the implementation of standards.	Large open-source databases Funding bodies requesting data standard and data sharing Rapid progression from foundational vaccine research to clinical application - Leveraging years of prior work on mRNA and viral vector vaccines, the first clinical trial for the COVID-19 vaccine started in mid-March 2020.
	Data in different language	Translation issue and lack of common terminology	
	Little data sharing, privacy issues	Strict GDPR^a^/HIPAA^b^ rules, government and hospital policies hampering data sharing, long process for DTA^c^ approvals	
Knowledge	Slow recognition of a novel pathogen	Lack of coordination between the different hospitals, open communication and information sharing	
	Lack of recognition of community spread		
	Lack of uniform global response		
	Poor communication with patients	Lack of evidence-based materials targeted to patients, lack of patient empowerment	
Practice	Lack of treatment selection guidelines	Little or no information on the possible treatment options and tools to assess novel therapies' efficacy and safety	Quick emergency use authorization FDA/EMA/WHO
	Lack of patient involvement in decision	Mistrust or disinformation regarding quarantine measures, effective therapies and vaccines	Principle of patient engagement and healthcare citizenship

a GDPR: General Data Protection Regulation

b HIPAA: Health Insurance Portability and Accountability Act

c DTA: Data Transfer Agreement

Despite the enthusiasm for the conceptual idea and early examples of successful LHSs, the synergy between healthcare practice and research, as was envisioned over a decade ago, is still far from reality [7]. A possible explanation for this gap between literature and practice is that the LHS policy proposals in the literature are very heterogeneous and the conceptual, legal, and practical aspects differ sensibly between countries and healthcare systems. Moreover, there are several levels of possible LHS implementation, which would require in turn more or less substantial interventions in terms of infrastructures, personnel training and mindset shift from clinicians, patients and authorities alike [6]. Another important issue to take into consideration is the current lack of interoperable FAIR (Findable, Accessible, Interoperable, and Reusable) data [15]. All these shortcomings are evident in a normal healthcare situation and even more so in an emergency like the COVID-19 pandemic. The use of predictive modelling based on AI could represent a viable approach to streamline the implementation of LHS practices in the healthcare systems, both on a national and international level and set the groundwork for a fully integrated LHS system that could support the management of future pandemics. Along with the shortcomings in the current pandemic management evidenced below, there have been also very positive aspects regarding for example the fast-track regulatory approval of new vaccines, off-label use of existing drugs and, at least in the US and Canada, emergency use authorization for medical devices [16], [17]. During the development of new drugs and vaccines during the pandemic, we witnessed, probably for the first time on such a large scale, the continuous integration of real-world data and clinical trial data, complementing each other in “real-time” [18], [19]. The proposed framework for the integration of AI and predictive modelling, based on clinical and imaging data, explores each pillar of the LHS and proposes some approaches to tackle future pandemics outbreaks.

## 4 Data – harmonization and standardization of clinical and imaging data

The foundation of an LHS lies in collecting and integrating data from diverse sources, such as EHRs, public health data, patient-generated data such as patient-reported outcome measures (PROMs), and research findings. This data would provide a comprehensive view of patient health, serving as a basis for generating actionable insights for individual patients and contributing to innovative and efficient research. During the pandemic, challenges hampered the realization of this potential: lack of data standardization and slow data sharing [20].

Data standardization refers to the implementation of a set of defined data elements, their characteristics and relationships, rules for creating, managing, and using the data elements, and a design that enables consistent collection and representation of these elements. Implementing such standards makes it easier to aggregate information and take advantage of the wealth of information for both research and management, which is also of utmost importance for an LHS to work effectively. To ensure that data are consistent, comparable, and can be integrated seamlessly across different sources, it is important to establish common data standards and practices such as common data models, controlled terminologies, and data exchange formats. Adopting FAIR data principles and Clinical Data Interchange Standards Consortium (CDISC) standardization can further facilitate the efficient collection, management, analysis, and reporting of clinical research data [21], [22]. By combining technical and organizational measures and through cross-sector collaboration, data standardization on a large scale can be achieved, thus enabling the development of a more effective LHS.

Nonetheless, sharing data during a pandemic can be slowed down due to legal and ethical aspects, such as the General Data Protection Regulation (GDPR) and the Health Insurance Portability and Accountability Act (HIPAA), which prioritize the protection of patient privacy. This can delay the development of state-of-the-art models during the early stages of a pandemic when much is still unknown [23]. To overcome these challenges, distributed learning systems are being increasingly implemented [24], [25]. Distributed learning is a general approach to developing machine learning (ML) models without the need to centralize the data and the model computations in a centralized server. This allows for respect of user privacy and brings ML to fields where data cannot be shared for different reasons. Among the different distributed learning approaches, federated learning allows each user to train part of the model locally. Each part of the model is then sent to the central server where the federated learning framework aggregates the different parts into a consistent model. This way, the data used to train the model never leaves the partner organization.

Federated learning has demonstrated significant promise in healthcare, particularly in synthesizing insights from Electronic Health Records (EHR) across various medical institutions. It enables the collaborative training of predictive models, addressing the challenge of limited patient data within individual hospitals and preserving patient privacy [26]. Notable applications include tensor factorization for phenotyping analysis, differentially private learning for EHR, and mortality rate prediction for heart disease patients without transmitting sensitive data [27], [28], [29]. Additionally, federated learning has been applied to natural language processing (NLP) tasks for processing clinical notes and biomedical imaging analysis, allowing for the extraction of features from data such as MRI scans while maintaining the confidentiality of patient information [29], [30], [31].

Moreover, federated learning has been applied to enhance COVID-19 screening using chest X-ray images. In a study by Feki et al., a federated learning framework enabled multiple medical institutions to collaboratively train deep learning models for detecting COVID-19 without centralizing sensitive patient data [32]. The framework effectively managed the innate challenges of unbalanced and non-independent and identically distributed (non-IID) data distributions, which are common in healthcare settings. The study demonstrated that models trained using federated learning could achieve comparable results to those trained in centralized settings, underlining the approach's viability for privacy-sensitive medical applications. This application is particularly salient, considering the urgent need for rapid and accurate diagnostics during a pandemic, and highlights federated learning as a tool for global collaborative efforts in public health crises.

However, it is crucial to acknowledge the limitations inherent in federated learning, particularly when applied to medical imaging. Variabilities in image acquisition protocols from different devices or sites, such as spatial resolution and slice thickness, pose challenges for model training, as federated learning relies on a degree of consistency among data sources [33]. These discrepancies pose challenges for model training, as federated learning assumes a level of consistency among the data sources. To address these challenges, computational data harmonization methods have been used to transform, standardise, aggregate, and match the multi-source data and match multi-source data, making it more amenable to federated learning systems [22]. Yet, even with these efforts, challenges in data quality, model convergence, and bias from non-representative local datasets remain. The non-independent and identically distributed (non-IID) nature of data across federation participants can further complicate model training, necessitating advanced algorithmic solutions to mitigate data distribution skew [34].

It would be beneficial to establish protocols and procedures for collecting data in a standardized and harmonized way on a continuous basis, ideally before a pandemic strike. By doing so, the resulting standardized data can be used to develop predictive models also in the early phases of the pandemic. This would also allow organisations to set up agreements in accordance with regulations such as GDPR and HIPAA, ensuring that the necessary legal and ethical protocols are already in place when a pandemic occurs.

## 5 Knowledge – Stakeholders communication and awareness

As high-quality data is collected and integrated, it must be transformed into actionable knowledge to inform clinical practice, allocation of medical resources, and public health interventions. The creation, dissemination, and application of evidence-based knowledge are critical in enabling effective and timely responses to disease outbreaks such as COVID-19. AI plays a pivotal role in supporting the knowledge pillar of LHS by unlocking valuable insights from vast amounts of data by enabling rapid analysis through techniques such as ML and NLP [35], [36], [37]. These techniques were instrumental in the accurate identification of COVID-19 cases and informing treatment decisions during COVID-19.

Models using imaging-based techniques can provide suggestive findings that aid in the clinical assessment of patients presenting with respiratory symptoms, helping to differentiate patterns often associated with various types of pneumonia, such as influenza, COVID-19, MERS, and "new" pneumonias. While definitive diagnosis of specific infections relies on molecular techniques, starting with metagenomic sequencing for novel pathogens, followed by bespoke nucleic acid tests for known pathogens, using ML algorithms and advanced image analysis techniques can help extract relevant features from medical images and classify them based on specific disease patterns. This can assist clinicians in prioritizing patients for further diagnostic testing and treatment. For example, deep learning methods were used to analyse chest CT scans to classify patients with COVID-19 [38]. In another study from the DRAGON consortium, an AI-based tool named CAD4COVID-CT was developed to detect COVID severity based on CT scans, enabling better prognosis and prediction of patient outcomes [39]. Another study developed a radiomics signature using ML techniques to automatically distinguish COVID-19 cases from other types of pneumonia based on CT scans [40]. The advantages of this approach are that it reduces the burden on clinicians and facilitates faster and more accurate diagnosis, both of which are crucial for an effective LHS in a pandemic.

Aside from diagnosis, AI/ML techniques also played a role in predicting mortality in COVID-19 patients. One study by Chatterjee et al. demonstrated the potential of AI in predicting COVID-19 mortality using demographic and comorbidity data, surpassing age-based predictions [35]. Such ML models, when integrated into an AI-enabled LHS, can contribute to more accurate risk stratification, allowing clinicians to prioritize resources and provide targeted care during a pandemic. Furthermore, the external validation of the models supports the generalizability of the authors’ findings, emphasizing the importance of data-driven approaches in clinical decision-making.

Furthermore, the integration of AI with geographic information systems (GIS) has provided significant advancements in the monitoring and management of infectious diseases. For example, one study used GIS alongside ML models to map the vulnerability of regions to COVID-19, taking into account factors such as population density, the percentage of older people, temperature, and humidity [41]. Their study applied multi-scale geographically weighted regression (MGWR) and an adaptive neuro-fuzzy inference system (ANFIS) to create predictive maps that can assist health officials in prioritizing interventions and resources. Such models demonstrate the potential of combining spatial analysis with AI to identify hotspots and forecast the spread of diseases, leading to informed and swift public health responses. These tools could help to visualize a pandemic’s progression and support decision-making in real-time by enabling the correlation of reported symptoms with specific locations.

In addition, NLP holds significant potential in an LHS in a pandemic setting. NLP involves automatically processing and analysing large amounts of unstructured textual data, such as electronic health records (EHR), scientific literature, and social media posts, which can help rapidly identify emerging trends, early detection of outbreaks, and real-time monitoring of the pandemic. NLP techniques were applied to address various challenges during the COVID-19 pandemic, including identifying potential therapeutic candidates, tracking misinformation, and monitoring public sentiment [42].

These studies exemplify the potential of AI-driven models and tools to transform data into valuable knowledge. However, it is also crucial to keep in mind the perspectives and needs of end-users such as clinicians, patients, policymakers, and members of the public when building the Knowledge pillar. The actionable knowledge generated from AI/ML-based data analysis must be communicated effectively to these stakeholders so that they can collaborate and make informed decisions based on the latest insights.

## 6 Practice – procedures and guidelines

The added value of predictive models and AI in the management of pandemics might be greatly reduced if the healthcare system is not ready to receive and implement the insights gained with these novel approaches. A fully integrated LHS also needs the support of taxpayers, the government, services providers and regulatory agencies. The healthcare ecosystem needs to be ready to apply and accept the use of routine care and patient monitoring of these innovative AI tools.

During a global pandemic such as COVID-19, an LHS could have played a pivotal role in delivering personalized care by predicting individual patient outcomes and triaging patients according to their risk levels. This requires the establishment of clear protocols, the identification of potential obstacles, and harmonization of efforts towards better leveraging 'big data' in healthcare. Open-source predictive and prognostic models based on AI/ML, such as those available at covid19risk.ai exemplify the type of innovation that can drive such an approach to managing pandemics and epidemics [43].

Authorities and policymakers must consider more agile regulatory processes that accommodate fast-track approvals for not just new drugs or vaccines but also medical devices, guidelines, and reimbursement strategies. The FDA, for instance, has an Emergency Use Authorization (EUA) pathway for medical devices, which saw extensive utilization during the COVID-19 pandemic [44]. Furthermore, the FDA has released draft guidance for continuous learning systems based on AI to be recognized as medical devices, underscoring the importance of AI and predictive modeling in a pandemic context [45], [46].

Another major roadblock to the implementation of these tools is the lack of trust among clinicians and patients. Trust in AI technologies is a significant barrier to their widespread acceptance in healthcare. In response to this challenge, the concept of Explainable AI (XAI) is gaining traction [47]. XAI aims to produce results understandable by humans, with processes that can be logically explained, in contrast to the 'black box' nature of some ML and AI systems. XAI algorithms must adhere to principles of transparency, interoperability, and explainability [48]. Clear regulatory guidelines and the advancement of XAI may help overcome some of the major hurdles to the broad adoption of predictive modeling in managing future pandemics, as outlined in Table 2.

**Table 2:** Predictive AI models for the management of future pandemics.

LHS pillars	Challenges	AI and predictive models
Data	No data standardization	CDISC data standards implementation across hospitals globally for distributed/federated data network
	Little data sharing	Distributed learning implementation globally to allow access to clinical data without concern about data sharing and GDPR/HIPAA
Knowledge	Slow recognition of a novel pathogen	Models for differential classification of different cases of pneumonia (Influenza, coronaviruses, MERS and novel pathogen) based on imaging and non-imaging data Models to monitor the increase in specific complaints, and increased hospital/general practitioner visits
	Lack of recognition of community spread	Automatic monitoring and correlation of locations that have cases with specific symptoms reported
	Lack of uniform global response	Warning system implementation to relevant authorities/regulatory bodies for a prompt response
	Poor communication with patients	Patient decision support apps where patients can share their symptoms and experiences and at the same time receive support and information on the most recent research results presented in layman’s terms
Practice	Lack of treatment selection guidelines	Imaging based tools to assess response to therapy, prognosticate disease development and inform clinical decision-making at the bedside

## 7 Recommendation on pandemic management and implementation of predictive modelling in future pandemics per stage of pandemic

The added value of AI and predictive models can be leveraged at each stage of a possible future pandemic, from the early detection phase up until the new therapeutic options monitoring. A virtuous cycle could be established with information gathered in previous steps, feeding development and validation of additional predictive models and approaches. Both the WHO and the Centers for Disease Control and Prevention (CDC) identified several influenza pandemic stages or phases and, while the guidelines have been developed before the COVID-19 pandemic, both recognize the need for continuous vigilance and cyclic evaluation of early pandemic signs, which fits in the concept and implementation strategy for LHS [49], [50]. In Table 3, we outlined the possible contribution of AI and predictive modelling with clear examples, along with the essential requirements in terms of data and processes that are necessary to implement and streamline these approaches. Far from being exhaustive, the summary would represent a starting point for discussion and for considering pandemic management with AI as a continuous and concerted effort more than a surge of activities in times of crisis.

**Table 3:** Role of AI and predictive models in future pandemic management.

Stage of pandemic	Requirements	AI and predictive modelling
Stage 0 – Before a new pathogen emerges	− Data harmonization and standardization − Distributed learning networks − Emergency regulatory approval procedures − Streamlined ethical approval for data sharing and clinical studies for new drugs/vaccines/medical devices in an emergency setting	• Models for differential classification of different cases of pneumonia (Influenza, coronaviruses and novel pathogens) integrated into hospitals globally or at least in areas where wildlife virus jump to human host is common such as areas of Asia (coronavirus, influenza, Nipah virus) and Africa (Zika, Ebola, HIV, etc.). • Models flagging significant increase in specific complaints, and increased hospital/general practitioner visits
Stage 1 – localized report of a new pathogen, unknown outcome or origin	− Patients' data - symptoms, timeline, imaging, disease outcome, therapies, demographics	• Early detection with multi-omics models (imaging, clinical and laboratory data)
Stage 2 – Reports of clusters and spread outside the country of origin	− Biological sample bank for future analysis (transcriptomics, genomics among others, to identify biomarkers associated with specific outcomes) − Follow-up clinical and imaging data	• Fast-track AI enhanced clinical trials for vaccine and therapeutics
Stage 3 – an exponential increase of cases reported – operation disruption of the healthcare system, crisis management	− Biological sample bank for future analysis (transcriptomics, genomics among others to identify biomarkers associated with specific outcomes) − Follow up clinical and imaging data − Robust data management and follow up for non-pandemic patients (such as cancer patients)	• Models predicting disease outcomes to help with patient triage and resource management • Patient monitoring tools for self-reported symptoms, outcomes and long-term effects (e.g., long-COVID) • Tools and apps allowing remote patient management for non-pandemic diseases to enable the standard of care for other patients.
Stage 4 – reduction of infection rate	− Viral genomic data on newly reported infections. − Analysis of previously collected biological samples	• Tools monitoring the emergence of new viral variants associated with distinct disease outcome
Stage 5 – Effective vaccine and/or treatment available	− Follow up clinical and imaging data (both recovered and immunized patients) − Consolidated real-world evidence for further drug/vaccine/devices development	• Companion diagnostic development aimed at specific patient cohorts – personalized vaccinology • Monitoring for breakthrough infections • Monitoring vaccine efficacy to determine the duration of protection and prepare for booster requirements

## 8 Conclusions

Despite the promise of AI and predictive modelling in pandemic management, the road ahead is still long. Authorities and policymakers need to implement strategies to promote the application of the LHS principles on a national and supra-national level. Acknowledging the strides made by exemplary healthcare systems can be a promising start. Certifying and accrediting medical centers, as well as regional and national healthcare systems, as "LHS compliant" may serve as a pivotal initial move to increase visibility and acceptance.

In the specific field of AI and predictive modelling, the main issue remains data sharing and access: building infrastructures and protocols, both technical and legal, to promote a “federated by design” approach could motivate clinical centres in putting effort to organize, standardize and share data, in a privacy preserving fashion.

Lastly, the current LHS model should move forward towards its central theme of community engagement, to become learning healthcare networks (LHN) [51], [52]. In this way, the LHN could focus on the provision of health care from the perspective of patients and communities. Supported by AI and predictive modeling, this approach can complement the healthcare system, truly equipping it for impending pandemics.

## Funding

This project has received funding from the Innovative Medicines Initiative 2 Joint Undertaking (JU) under grant agreement No 101005122. The JU receives support from the European Union’s Horizon 2020 research and innovation programme and EFPIA.

## CRediT authorship contribution statement

**Philippe Lambin:** Funding acquisition, Project administration, Supervision, Writing – review & editing. **Guang Yang:** Formal analysis, Validation, Writing – original draft, Writing – review & editing. **Wim Vos:** Project administration, Supervision, Writing – review & editing. **Simon LF Walsh:** Formal analysis, Validation, Writing – review & editing. **Xiaodan Xing:** Formal analysis. **Yang Nan:** Formal analysis. **Rebecca Baker:** Conceptualization, Validation, Writing – original draft, Writing – review & editing. **Kit Howard:** Conceptualization, Investigation, Validation, Writing – original draft, Writing – review & editing. **Inês Freitas Pinho:** Conceptualization, Investigation, Project administration. **Fabio Bottari:** Conceptualization, Formal analysis, Methodology, Visualization, Writing – original draft, Writing – review & editing. **Lisanne Eppings:** Conceptualization, Formal analysis, Writing – original draft, Writing – review & editing. **Anshu Ankolekar:** Conceptualization, Formal analysis, Methodology, Visualization, Writing – original draft, Writing – review & editing.
